# Extremely Rare Co-occurrence of Left Gastroschisis-Like Abdominal Wall Defect and an Omphalocele in a Very Low Birth Weight Infant: A Case Report

**DOI:** 10.70352/scrj.cr.25-0453

**Published:** 2025-10-29

**Authors:** Teizaburo Mori, Hirofumi Tomita, Ayano Tsukizaki, Kazuki Hirohara, Ayano Hidaka, Nobuhiro Takahashi, Akihiro Shimotakahara, Fumiya Okazaki, Keita Osumi, Kaoru Okazaki

**Affiliations:** 1 Department of Surgery, Tokyo Metropolitan Children’s Medical Center, Fuchu, Tokyo, Japan; 2 Department of General Pediatrics, Tokyo Metropolitan Children’s Medical Center, Fuchu, Tokyo, Japan; 3 Department of Neonatology, Takatsuki General Hospital, Takatsuki, Osaka, Japan; 4 Department of Neonatology, Tokyo Metropolitan Children’s Medical Center, Fuchu, Tokyo, Japan

**Keywords:** gastroschisis, omphalocele, artificial patch, hydro-fiber dressing coating, staged operation, component separation

## Abstract

**INTRODUCTION:**

Gastroschisis is almost always a small, right-sided, periumbilical, abdominal wall defect, and its occurrence on the left side of the umbilicus is extremely rare. Furthermore, omphaloceles and gastroschisis usually do not co-occur. The present report is the 2nd worldwide to describe the co-occurrence of an omphalocele and left gastroschisis-like abdominal wall defect.

**CASE PRESENTATION:**

A 32-year-old pregnant woman (gravidity 2 and parity 1) was referred to our center because gastroschisis was detected in her fetus at 15 weeks and 3 days of gestational age. At 33 weeks and 3 days of gestational age, cesarean section was performed to deliver the female infant after a premature rupture of membranes. Her birth weight was 1368 g, and her Apgar scores were 8 at 1 min and 9 at 5 min. At birth, a macroscopic examination revealed an omphalocele with liver prolapse and a large, left-sided, epigastric, abdominal defect with an associated prolapse of the liver, spleen, stomach, and intestine. The patient had 2 very challenging conditions, namely, an early delivery with immature lung function and 2 large abdominal defects, for which a staged operation was performed using a combination of techniques involving the application of an artificial patch to close the abdominal wall temporarily, a hydro-fiber dressing to promote epithelization, and component separation to close the rather large defect permanently. Contrast CT revealed a defect in the lower costal cartilage and hypoplasia of the upper left abdominal wall. The peripheral part of the left superior epigastric artery was unclear in the imaging study.

**CONCLUSIONS:**

The abdominal wall defect might have differed from other cases of left-sided gastroschisis in terms of its pathogenesis in the upper left abdomen. The presence of lower left hypoplastic thorax strongly suggested that dysfunction of the left superior epigastric artery had caused the abdominal wall defect. There is no standard treatment for this extremely rare, congenital malformation. Thus, its treatment requires the application of several techniques, each to address different aspects of the condition at the corresponding, surgical stage.

## Abbreviation

HFNC
: high flow nasal cannula

## INTRODUCTION

Gastroschisis is a congenital anomaly caused by the failure of the anterior abdominal wall to close completely during development, resulting in the release of the viscera into the amniotic fluid.^1)^ Gastroschisis is clinically characterized as a small, right-sided, periumbilical, abdominal wall defect, and its occurrence on the left side of the umbilicus is extremely rare. Left-sided gastroschisis is predominantly seen in female patients and is often associated with extra-intestinal anomalies.^2)^

An omphalocele is a congenital, midline, abdominal wall defect involving the herniation of the intra-abdominal organs into a membrane consisting of the internal peritoneum and the external amnion.^3)^ Omphaloceles and gastroschisis rarely co-occur.^4)^

The present report is the 2nd worldwide to describe left gastroschisis-like abdominal wall defect co-occurring with an omphalocele. The patient had 2 challenging conditions, namely, an early delivery with immature lung function and 2 large, abdominal defects, which were treated using a staged operation involving various techniques, such as the application of an artificial patch to close the abdominal wall temporarily, a hydro-fiber dressing to promote epithelization,^5)^ and component separation to close the defect permanently.^6)^ The present report describes the details of the treatment course and discusses the pathophysiology of this rare condition.

## CASE PRESENTATION

A 32-year-old pregnant woman (gravidity 2 and parity 1) was referred to the study center after gastroschisis was detected in her fetus at the gestational age of 15 weeks and 3 days. She was healthy and had no history of smoking or alcohol consumption. An ultrasound examination of the fetus found no other abnormalities, and a chromosome analysis of the amniotic fluid found a normal 46XX pattern. MRI performed at 31 weeks of gestation demonstrated that the liver, spleen, stomach, small intestine, and colon had prolapsed into the amniotic fluid and suggested that part of the liver might have prolapsed into the umbilical cord.

**Fig. 1:**
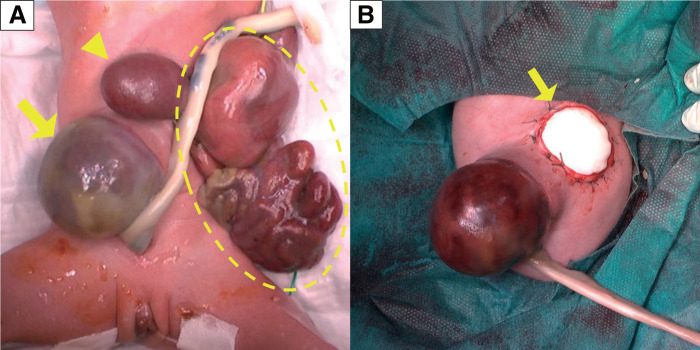
Macroscopic findings at birth and the end of the 1st operation. (**A**) Abdominal findings at birth. The intestine was found encapsulated and adhering to the surrounding tissue (dotted circle). The change in the internal color suggested a perforation. The lateral segment of the liver had prolapsed (arrowhead). An omphalocele was diagnosed postnatally (arrow). (**B**) Findings at the end of the 1st operation. The lateral segment of the liver was used to fill the left gastroschisis-like abdominal wall defect, and the defect was covered with an artificial patch (arrow).

A cesarean section was performed to deliver the female infant at 33 weeks and 3 days of gestational age following premature rupture of membranes. Her birth weight was 1368 g, and her Apgar scores were 8 at 1 min and 9 at 5 min. At birth, a macroscopic examination revealed an omphalocele and a large, left-sided, epigastric defect with prolapse of the liver, spleen, stomach, and intestines (**Fig. 1A**).

The 1st operation was performed on the day of birth to treat the prolapse of the viscera through the abdominal wall defect. The intestines formed an inflammatory, adhesive mass. After the adhesion was dissected, multiple, small, intestinal perforations were found. The left lobe of the prolapsed liver strongly adhered to the edge of the defect, which was adjacent to the hepatic vein and the left diaphragm. As difficulty was encountered when attempting to insert a silo patch into the abdominal cavity through the defect, the perforated intestine was resected, and the residual intestine was anastomosed to provide luminal continuity. After returning all the prolapsed organs into the abdominal cavity, the wall defect was covered with a soft tissue patch (Gore-Tex Soft Tissue Patch; W.L. Gore & Associates, Newark, DE, USA) (**Fig. 1B**).

**Fig. 2:**
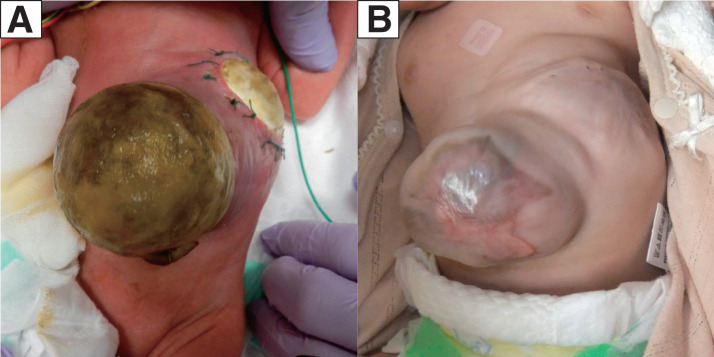
Findings after the 1st operation. Epithelialization using a hydro-fiber dressing containing silver. (**A**) Postoperative week 2 and (**B**) postoperative month 6.

The abdominal wall defect areas were postoperatively covered with a hydro-fiber dressing containing silver (Aquacel Ag; Convatec, London, UK) to prevent infection and drying. When signs of infection were detected on postoperative day 46, the patch was removed to reveal a fibrin layer that had formed beneath it. Epithelialization was achieved by 4 months of age with continued use of hydro-fiber dressings (**Fig. 2**). After a depression in the lower thorax was found postoperatively, the patient was discharged for a time at 5 months of age with an HFNC for respiratory support.

**Fig. 3:**
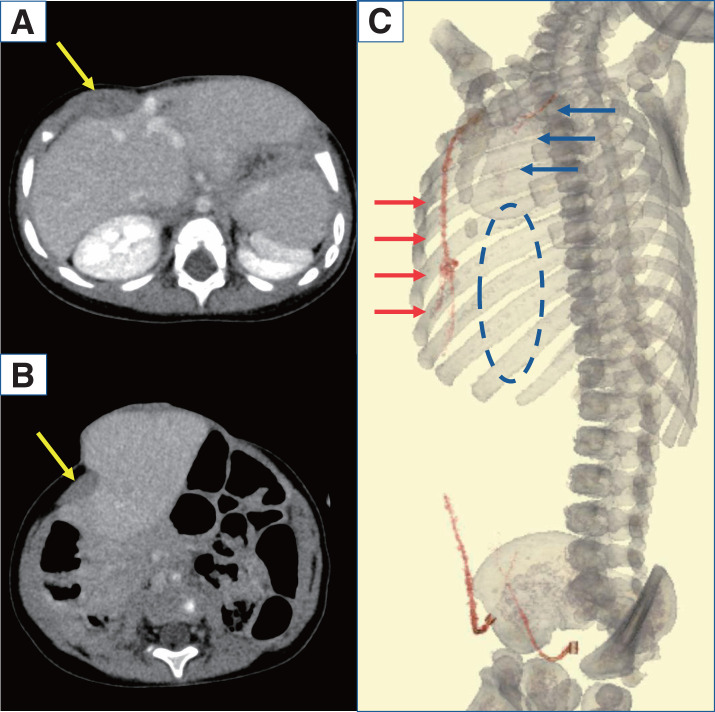
Contrast CT before the 2nd operation. Contrast CT images taken before the 2nd operation. On the axial plane, the rectus abdominis muscle can be seen on the right (arrow) but is absent on the left (**A**, **B**). On vascular reconstruction imaging, the area from the internal thoracic artery to the superior epigastric artery was able to be confirmed on the right side (red arrow). On the left side, the internal thoracic artery appeared thin (blue arrow), and the presence of the superior epigastric artery was not able to be confirmed (blue dotted circle). The inferior epigastric arteries were identifiable on both sides (**C**).

The 2nd operation was performed at 10 months of age to treat the omphalocele. Preoperative contrast CT revealed a defect in the lower costal cartilage and hypoplasia of the upper left abdominal wall. The right-sided rectus abdominis muscles were normal, but the upper left rectus abdominis muscles were absent (**Fig. 3A** and **3B**), and the peripheral part of the left superior epigastric artery was unclear in the imaging study (**Fig. 3C**).

The 2nd operation removed epithelialized, skin-like tissue from the omphalocele. The abdominal wall was extended by applying the component separation technique on the right side. In the upper left part of the abdominal wall, fascial tissue, the only sturdy tissue found, was sutured to the right rectus abdominis.

**Fig. 4:**
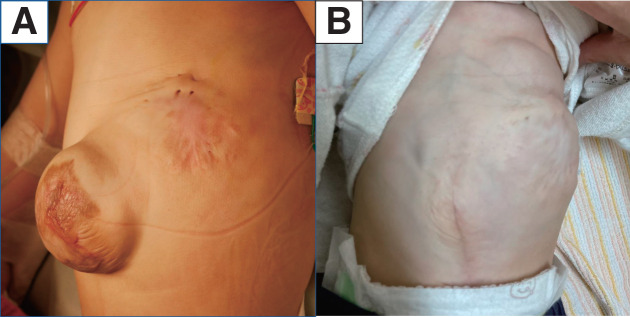
Findings after the 2nd operation. (**A**) Findings at the time of the omphalocele repair surgery and (**B**) 8 months after the 2nd operation.

The patient’s postoperative course was uneventful. Respiratory support ceased to be necessary at postoperative month 1 (**Fig. 4**). Currently, the patient periodically visits the outpatient clinic for routine follow-up examinations.

## DISCUSSION

Gastroschisis is a rare defect of the abdominal wall having an incidence rate of 0.3–1 per 10000 births.^7)^ Left-sided gastroschisis, first described by Blair and Marshall in 1988,^8)^ is a much rarer, congenital malformation. To the best of our knowledge, only around 35 cases have been reported in the English language literature, and approximately half the cases involved complications.^2,4)^ By contrast, only 14% of cases of right-sided gastroschisis described to date involved complications.^9)^ In most cases of left-sided gastroschisis, a small, abdominal wall defect occurs near the umbilical cord, but in some cases a large defect may be found in the upper left abdomen, which is described as “an unusual, left-sided, abdominal wall defect” by several sources.^10,11)^

Omphaloceles and left-sided gastroschisis rarely co-occur. To the best of our knowledge, only one such co-occurrence, of which the phenotype closely resembled that of the present case, has thus far been reported.^4)^ In both cases, the left-sided gastroschisis involved a large defect of the upper left abdomen.

The pathogenesis of gastroschisis, particularly its molecular mechanism, has yet to be fully elucidated. Many theories have been proposed^12)^; the most generally accepted theory holds that the umbilical vein regresses, leaving a weakened fascial region through which the abdominal contents herniate.^13)^ An alternative theory posits an early disturbance of the omphalomesenteric artery which leads to the fascial weakness.^14)^ If either of these theories is correct, regression of the left umbilical vein or left omphalomesenteric artery might lead to left-sided gastroschisis characterized by a small, abdominal defect near the umbilicus.^11,15)^

In the present case, contrast-enhanced CT performed before the 2nd operation indicated a hypoplastic pattern in the left superior epigastric artery. The impairment of the blood supply to this area might have caused the anatomical hypoplasia of the lower left costal cartilage and the abdominal wall defect. These findings support the etiological hypothesis of impaired blood flow as the cause of the abdominal wall malformation.

Left- and right-sided gastroschisis usually do not differ in terms of treatment. In a previously reported case of co-occurrence of an omphalocele with left gastroschisis-like abdominal wall defect, the latter was initially treated with a silo bag while the omphalocele was treated with topical agents until complete epithelialization was achieved.^4)^

The left, epigastric wall defect in the present case was large and accompanied by severe inflammation. Because creating a silo to cover the prolapsed organs proved difficult, an artificial patch was used for this purpose. The increase in the abdominal pressure following the replacement of the prolapsed organs to the abdominal cavity increased the distention of the large omphalocele. Recent studies have demonstrated that under such conditions, various tools, such as hydro-fiber dressings and antiseptic cream,^5,16)^ may be effective in promoting epithelialization as part of a conservative treatment strategy. Indeed, the hydro-fiber dressings used in the present case had the desired effect of promoting epithelialization quite well. The final stage of the treatment, aimed at closing the omphalocele, involved the use of component separation, which was sufficient to treat the condition despite being applied only to the right side.

## CONCLUSIONS

The present report described the treatment of an extremely rare co-occurrence of left gastroschisis-like abdominal wall defect and an omphalocele. The large, abdominal defect was located in the upper left abdomen and may have differed etiologically from the only other instance of left-sided gastroschisis reported thus far; hypoplastic changes observed in the left lower thorax strongly suggested that a disturbance of the blood flow in the left superior epigastric artery had caused the abdominal wall defect. As there is no standard treatment for this extremely rare, congenital malformation, it is important to apply a method that is appropriate to each stage of its treatment.

## DECLARATIONS

### Availability of data and materials

All the related data are included with the article.
